# Artificial intelligence anxiety: a narrative review of psychiatric conceptualization and clinical management

**DOI:** 10.12701/jyms.2026.43.54

**Published:** 2026-08-10

**Authors:** So Hye Jo

**Affiliations:** Department of Psychiatry, Yeungnam University College of Medicine, Daegu, Korea

**Keywords:** Anxiety, Artificial intelligence, Mental health, Psychological adaptation, Psychological stress

## Abstract

Artificial intelligence (AI) is gradually permeating every aspect of daily life. AI is now more than a mere tool; it significantly influences individual capabilities, professional values, social status, and our perspectives on the future. Amid these AI-driven changes, the concept of AI anxiety (AIA) has emerged to describe the associated emotional distress; however, its psychiatric significance has not yet been clearly defined. Presently, AIA can be conceptualized not as a distinct psychiatric diagnosis, but as a stress response capable of amplifying pre-existing vulnerabilities, psychiatric symptoms, maladaptive coping mechanisms, and functional impairments. This review categorizes AIA into five interrelated domains: anxiety regarding competence and adaptation, anxiety concerning occupational displacement and role loss, anxiety related to sociotechnical mistrust and loss of control, anxiety regarding identity and the human-machine boundary, and existential or catastrophic anxiety regarding the future. The clinical assessment of patients should evaluate specific AI-related experiences, perceived threats, behavioral responses, functional consequences, comorbid symptoms, and reality-testing abilities. Cases accompanied by excessive reliance on AI, social isolation, or the substitution of human support with AI interactions warrant increased attention. Therapeutic approaches include psychoeducation, cognitive and behavioral strategies, acceptance-based approaches, meaning-centered psychotherapy, AI literacy education, and efforts to restore human connections. Future studies should clarify the definition and scope of AIA and elucidate its course and treatment responsiveness through longitudinal and clinical studies using reliable assessment tools.

## Introduction

Artificial intelligence (AI) technology is advancing rapidly. Its integration into various fields such as healthcare, education, and industry is rapidly transforming our social environments and daily lives [1]. These changes represent unprecedented opportunities and challenges for human society. Specifically, generative AI (GenAI) has advanced to a level where it mimics, or even surpasses, human capabilities such as creativity, reasoning, and linguistic communication, which were once considered uniquely human traits. These technological advancements have elicited complex emotional responses from humanity [2,3]. As AI has evolved beyond mere technological tools to exert a massive, powerful influence that reshapes jobs, identities, and future prospects, the term AI anxiety (AIA) has emerged [4]. AIA is not limited to specific occupational groups or those with limited technical skills; it is expanding into a universal psychological response that occurs widely across all ages, occupations, and employment status [5]. This highlights the need to address it as a social-level mental health issue stemming from technological advancements and the resulting societal changes that affect individuals’ mental health, including human identity and perceptions of oneself and the future. From this perspective, the need for attention and intervention from a psychiatric standpoint is further emphasized [6,7].

Although AIA is an emerging concept attracting increasing research attention, it has not yet been clearly defined. Furthermore, studies directly addressing this topic are limited. Specifically, research conceptualizing AIA from a psychiatric perspective and discussing it as a subject of clinical assessment and intervention is insufficient.

This narrative review examines the literature, focusing on key terms such as AIA, technostress, AI literacy, job replacement anxiety, AI chatbots, GenAI, and mental health [6,7]. This review aimed to comprehensively synthesize AIA-related concepts and evidence, which have been scattered across various sources to date, from a psychiatric perspective. The content emphasizes the conceptualization of AIA, clinical assessments, potential interventions, limitations, and future directions.

## Conceptualization of artificial intelligence anxiety

### 1. Definition of artificial intelligence anxiety

AIA can be broadly defined as a state of fear, anxiety, and stress stemming from a lack of sufficient understanding or control over the potential effects and development of AI technology [2,5,6]. Although AIA is partially distinct from technostress, it represents a different strain [8,9]. Technostress refers to stress arising primarily from the use of information and communication technologies, including techno-overload, techno-intrusion, techno-complexity, techno-anxiety, and techno-uncertainty [10]. By contrast, AIA originates not from individual users but from the inherent nature of AI. Factors such as autonomous decision-making, unpredictability, lack of choice, privacy concerns, direction of choices, and implications for human existence are associated with AIA. Notably, these factors tend to provoke greater anxiety because they lie beyond the direct control of individual users [5,11]. Compared with traditional technostress, the perception of threats to human agency, professional roles, or social values can trigger a deeper form of existential anxiety.

Johnson and Verdicchio [4] argued that AIA is fueled by misunderstanding and confusion regarding AI, particularly an excessive focus on autonomous AI systems without considering human involvement, confusion regarding autonomy in computational entities, and inaccurate conceptions of technological development. Subsequently, Li and Huang [11] expanded the concept of AIA and proposed eight subfactors: anxiety about privacy infringement, biased behavior, job displacement, learning, existential risk, ethical violations, artificial consciousness, and lack of transparency. They interpreted and explained the pathways leading to AIA within the framework of integrated fear acquisition theory. AIA was explained as consisting of four main categories: anxiety arising from direct experience with AI (conditioning), anxiety arising from seeing or hearing about others being harmed by AI (vicarious exposure), anxiety arising from learning about the risks of AI through various media (transmission of information/instruction), and primal anxiety stemming from the uncertainty of AI (inability to recall a pertinent experience).

### 2. Psychiatric reconceptualization of artificial intelligence anxiety

When treating patients with AIA from a psychiatric perspective, the focus of clinical assessment and intervention must vary according to the specific AIA subfactors. Consideration should include how each AIA subdimension is experienced as a psychological threat by the individual and through which psychological mechanisms it transforms into anxiety. Accordingly, this study reclassifies AIA from a psychiatric perspective and conceptualizes it into five categories: competence and adaptation anxiety, anxiety regarding occupational and role loss, anxiety regarding sociotechnical distrust and loss of control, identity anxiety and human–machine boundary anxiety, and existential and catastrophic future anxiety (Fig. 1) [8,11,12].

#### 1) Competence and adaptation anxiety

Competence and adaptation anxiety refers to the anxiety individuals feel about their learning and adaptive abilities amid the rapid advancement of AI technology. This category relates to “learning anxiety,” a subdimension of AIA, and refers to the difficulties and anxiety associated with understanding, learning, and utilizing AI technologies [5,11]. The feeling of being unable to keep pace with a rapidly changing environment lowers self-efficacy, hinders task performance, and contributes to negative expectations regarding future adaptation and feelings of helplessness [13]. Specifically, because AI technology is rapidly integrating into all aspects of work, education, communication, and information processing, individuals may experience a sense of threat in that their existing competencies are no longer sufficient. A previous study on AI literacy has suggested that the ability to understand, use, evaluate, and ethically engage with AI can enhance AI-related self-efficacy and reduce anxiety in AI-supported learning contexts [14]. Although this anxiety can be viewed as an adaptive signal that motivates individuals to acquire new skills, excessive anxiety can lead to avoidance, social withdrawal, disinterest in learning, and self-blame.

#### 2) Occupational displacement and role-loss anxiety

Occupational displacement and role-loss anxiety stems from the perception that AI can replace human labor or undermine an individual’s professional values. This category corresponds closely to the “job replacement” dimension of AIA identified in previous multidimensional models of AIA [5,15]. From a psychiatric perspective, this extends beyond concerns about economic loss to threats to professional identity, social roles, and self-worth. In modern society, a job serves not only as a source of income, but also as a fundamental element shaping an individual’s identity, social belonging, perceived competence, and future plans [16]. Therefore, the possibility of AI replacing one’s job may be experienced not merely as a fear of unemployment but also as an ontological and social threat, the fear that one’s role may no longer be needed.

Therefore, AI awareness may function as a workplace stressor by increasing job insecurity and emotional exhaustion [14,17]. Such anxiety can be linked to job insecurity, depression, anxiety, helplessness, and occupational burnout and may vary according to career stage, occupational field, task characteristics, and perceived replaceability.

#### 3) Sociotechnical distrust and loss-of-control anxiety

Sociotechnical distrust and loss-of-control anxiety stems from concerns that AI systems may monitor individuals or make unexplainable decisions. This category includes anxieties regarding privacy violations, biased or discriminatory behavior, and a lack of transparency [5,11]. From a psychiatric perspective, these three subdimensions share a common psychological mechanism: a loss of predictability and a sense of control. This interpretation aligns with the broad body of psychological literature suggesting that a reduced sense of control and intolerance to uncertainty are key mechanisms underlying anxiety responses [18-20]. This anxiety may intensify as AI plays an increasingly substantial role in decision-making across key areas of life such as employment, finance, healthcare, education, justice systems, and public administration. Such apprehension can be particularly difficult for individuals with low tolerance of uncertainty.

#### 4) Identity and human-machine boundary anxiety

Identity and human-machine boundary anxiety arises when AI is perceived as more than a mere tool, rather as an entity possessing human-like consciousness, emotions, judgment, and moral agency. This category includes anxiety about artificial consciousness and ethical violations, which were identified as AIA subdimensions in previous work [11]. AIA distinctly involves experiences in which the boundary between humans and nonhumans becomes blurred. Conversational AI, social robots, and anthropomorphized AI services respond similarly to humans, appear to empathize, and can sometimes be experienced as relational entities. This is consistent with the “computers are social actors” paradigm, which suggests that individuals often apply social rules and expectations to computers and interactive media despite knowing that they are not human [21].

Consequently, individuals may be confused about whether AI is a tool or an independent agent. From a psychiatric perspective, this is associated with threats to human uniqueness, confusion about boundaries, anthropomorphism, and alienation [22-25].

#### 5) Existential and catastrophic anxiety about the future

Existential and catastrophic anxiety about the future stems from the perception that advancement of AI could threaten human survival and the safety of future society. This category corresponds to “anxiety about existential risks,” which is one of the existing subdimensions of AIA [11]. From a psychiatric perspective, this category reflects distress related to large-scale future threats that remain challenging to predict, influence, and control. Such distress may be reinforced by negative cognitive biases, a tendency to interpret uncertain events in catastrophic terms, and a low tolerance of uncertainty [20,26,27].

When AI is perceived as potentially surpassing human intelligence, developing with increasing autonomy, and becoming challenging to regulate, uncertainty about whether it can remain under human control may produce persistent but poorly defined fears of the future [28,29]. These fears do not arise solely from direct encounters with AI; they may also be strengthened by imagined scenarios, public debates, media narratives, and warnings from prominent experts [30,31].

Although the five aforementioned categories are conceptually distinct, they can influence each other. For instance, anxiety about failing to adapt to rapid AI advancements can amplify the fear of losing one’s job to AI. Similarly, the distrust of AI systems can lead to broader anxieties regarding the future and survival of human society. Therefore, AIA can be conceptualized as a multidimensional psychological response, characterized by a diminished sense of human agency, that arises when individuals perceive their social roles, autonomy, and enduring value as human beings to be threatened by rapidly evolving AI technology. Therefore, AIA treatment must extend beyond merely alleviating anxiety symptoms and focus on restoring an individual’s sense of agency, control, and meaning in life [5,11].

### 3. Risk factors and vulnerable populations

The intensity and manifestations of AIA may vary depending on an individual’s demographic characteristics and socioeconomic status. These factors provide important clues for identifying clinically vulnerable groups. AIA severity varies significantly according to sex, age, educational level, employment status, and occupational status.

With regard to sex, a large-scale study found that women reported higher levels of AIA than men [32]. However, since sex differences may vary depending on the cultural context, occupational group, experience with technology, and educational level, they should be understood within a social and occupational context rather than interpreted as simple biological differences.

AIA was particularly high among older adults aged ≥60 years and retirees. This suggests that the digital divide, reduced technological familiarity, and diminished self-efficacy regarding new technological environments have been major contributors to high AIA [33]. However, AIA is not limited to older adults. Young adults who frequently use GenAI are also experiencing AI-related anxiety with increasing frequency. For this group, anxiety is more likely to be driven by job insecurity, uncertain career prospects, and fear of becoming less competitive in the AI-driven labor market [34]. This finding indicates that AIA should not be limited to specific age groups. Its manifestations may vary across the life cycle, reflecting developmental needs, occupational pressures, and social roles characteristic of each life stage.

An individual’s attitude toward AI is significantly influenced by the level of digital literacy. AI literacy encompasses the ability to understand how AI works, utilize it flexibly, efficiently evaluate AI-generated outputs, and understand their social implications. Without such literacy, individuals may perceive AI as unpredictable and difficult to control, potentially resulting in AIA. A recent study found a significant negative correlation between AI literacy and AIA [35]. Therefore, AI literacy education is essential to help individuals evaluate AI-related risks from a balanced perspective and effectively adapt to technological change [36].

Employment status and occupational group are also significant risk factors for AIA. Individuals engaged in simple, repetitive tasks where job security is low or the likelihood of AI automation is high, may experience greater anxiety regarding job displacement [5]. However, AIA is not limited to individuals engaged in simple and repetitive tasks. Recent reports have described anxiety and a sense of threat related to the expanding capabilities of GenAI, even in fields that have traditionally depended on human creativity, judgment, and expertise such as writing, design, arts, translation, education, healthcare, and law [37,38].

### 4. Clinical implications for psychiatric practice

AIA has not been established as an independent psychiatric diagnosis. Even under a conservative interpretation of the available evidence, the causal links between AIA and psychiatric symptoms remain limited. Therefore, it is difficult to determine whether AIA is the direct cause of specific mental disorders. Rather, AIA is more appropriately viewed as a psychosocial stressor that, through interactions with factors such as rapid sociotechnological changes, individual vulnerabilities, occupational environments, patterns of information exposure, social isolation, and impaired reality testing, can trigger symptoms of anxiety, depression, and obsessive-compulsive disorder, as well as functional impairment. Emerging evidence has suggested that AI-related stress is associated with emotional symptoms. In a cross-sectional study of 217 Romanian adults, AI-related technostress was associated with anxiety (β=0.342, *p*<0.001; R²=0.117) and, to a lesser extent, depressive symptoms (β=0.308, *p*<0.001; R²=0.095) [39]. However, the cross-sectional design and use of a general technostress measure rather than an AIA-specific scale preclude causal interpretation. Accordingly, clinicians should adopt an approach that facilitates the early assessment of symptoms, functional impairment, and high-risk signs in individuals with AIA.

### 5. Psychiatric assessment of artificial intelligence anxiety

Semi-structured clinical interviews are useful for identifying the characteristics of AI-related stressors in a patient’s life and their effects. This assessment should examine how AI-driven technological changes affect the patient’s emotional well-being, self-perception, future aspirations, professional identity, and interpersonal functioning. Questions such as “How have recent advancements in AI affected your daily life?” “What are your greatest concerns regarding an AI-driven future?” and “Have you ever felt that your professional role or value might be diminished due to AI?” can help identify the key features of AIA. Furthermore, the assessment must go beyond simply determining whether a patient uses AI to encompass the patterns and context of that usage. Relevant factors include frequency and duration of use, motivations for using AI, emotional reactions during and after use, and repetitive or difficult-to-control behaviors related to searching for AI-related information.

Moreover, determining whether AI use serves as a means of seeking reassurance or emotional avoidance is particularly important. Although AI-based tools and chatbots can offer temporary emotional support, caution is warranted. For some psychologically vulnerable patients, these tools may increasingly replace relationships with human support figures. Such substitutions can exacerbate interpersonal withdrawal, diminish willingness to seek help, and potentially worsen symptoms. Therefore, when a patient relies excessively on AI chatbots for emotional support, clinical assessments must carefully evaluate signs such as avoidance of face-to-face relationships, resistance to engaging in treatment, deepening social isolation, and disengagement from professional or informal care.

A representative tool for specifically quantifying AIA is the Artificial Intelligence Anxiety Scale (AIAS) [5]. The AIAS is a self-report scale consisting of a 7-point Likert scale. A higher total score indicates greater AIA severity. This scale evaluates AIA across four dimensions: learning, job displacement, sociotechnical blind spots, and AI composition. The sociotechnical blind spot dimension includes concerns regarding the limitations of human dependence on AI systems and the potential for human exclusion resulting from AI adoption. The AI composition dimension reflects fears about human-like AI. Technostress-related scales can also be used as supplementary tools for assessing technology-related stress. The technostress creators scale assesses key factors of technostress such as technology overload, intrusion, complexity, instability, and uncertainty [40]. Although this scale does not directly measure AIA, it helps identify the aspects of technology-related stress that are closely linked to patient anxiety. These aspects include the excessive learning required for new technologies, the encroachment of work into personal life, the burden of mastering unfamiliar systems, the pressure of constant software updates, and the psychological distress caused by the unpredictability of technological changes.

AIA should be assessed along with overall psychiatric symptoms. Standardized symptom assessment tools can help to determine whether AI-related concerns are accompanied by clinically significant anxiety, depression, or stress. The Generalized Anxiety Disorder-7 scale is used to quantify generalized anxiety symptoms, and the Patient Health Questionnaire-9 assesses the severity of depressive symptoms. The Depression, Anxiety, and Stress Scale-21 is particularly useful when all three symptom domains (depression, anxiety, and stress) need to be evaluated using a single instrument. These assessment tools provide a baseline for tracking changes in symptom burden over time and monitoring treatment responses.

Moreover, a clinical assessment is necessary to make a medical judgment that distinguishes between valid concerns and anxiety rooted in psychopathology. The clinician must determine whether the patient’s fear is based on realistic occupational, social, or technical risks or if it has evolved into a state that is persistently excessive and disproportionate to the actual threat. Additionally, the clinician should assess whether the patient is avoiding AI-related tasks, workplaces, information, and social situations.

In addition, the evaluation process must assess whether reality testing is impaired. Clinicians must assess whether concerns regarding AI surveillance, manipulation, replacement, or control are based on valid grounds or have become fixed, unrealistic, and challenging to resolve. Finally, the clinical significance of AIA must be established by reviewing its consequences such as decreased occupational or academic performance, sleep disturbances, social withdrawal, reduced engagement in treatment, social isolation, and suicidal thoughts or behaviors.

### 6. Psychiatric management of artificial intelligence anxiety

Clinical interventions for AIA focus on helping patients understand the threats associated with technological change more realistically, regulate their emotional responses to uncertainty, and rebuild healthy relationships with AI. As AIA has not yet been established as a distinct diagnostic category, the therapeutic approach should be based on a clinical formulation that comprehensively considers the patient’s symptom presentation, functional impairment, underlying mental disorders, AI exposure, and occupational and social stressors, rather than on standardized treatment protocols for a specific disorder.

The goals of psychiatric intervention can be summarized into four main points: (1) to develop a realistic and balanced assessment of AI; (2) to help individuals regain a sense of control and self-efficacy; (3) to reestablish identity, human uniqueness, and the meaning of life, which have been shaken by AI; and (4) to adjust digital habits so that AI use and information exposure do not interfere with the patient’s sleep, emotions, interpersonal relationships, or therapeutic alliances. From this perspective, AIA interventions may include the following approaches (Table 1).

#### 1) Psychoeducation

Psychoeducation explains that AIA is not a technical rejection or an overwhelming fear, but a stress response to a rapidly changing sociotechnical environment. It helps patients accept distress as a normal phenomenon. Moreover, it explains maladaptive coping behaviors, such as avoidance (which may result from excessive anxiety), as well as sleep disorders, depression, and social isolation, thereby helping patients cope effectively with these issues [6]. This psychoeducational approach helps patients recognize AI not as an uncontrollable external threat but as one of the many tools they can understand, manage, and utilize.

#### 2) Cognitive behavioral therapy

Cognitive behavioral therapy (CBT) is a primary treatment method that helps reduce symptoms such as depression and anxiety by training patients to understand and modify the cycle of thoughts, emotions, and behaviors. This therapy addresses catastrophic thinking, overestimation of probability, dichotomous thinking, selective attention, and confirmation bias observed in AIA [41,42]. People with AIA tend to perceive it as an absolute threat to their survival, career, values, and interpersonal relationships [5]. Thoughts such as “AI will replace my job” or “No matter how hard I try, I cannot keep up with AI” are typical core automatic thoughts that perpetuate anxiety.

Therapeutically, CBT involves identifying the patient’s automatic thoughts, exploring evidence supporting those thoughts, and helping the patient consider alternative interpretations [43,44]. For example, the thought that “AI will completely replace my job” can be reframed into a more balanced perspective, such as “Although AI may automate some tasks, my judgment, relationship management, and the weight of ethical responsibility remain crucial.” Through this cognitive restructuring, which helps individuals perceive AI not as an overwhelming threat but as part of an adaptable environmental change, anxiety can be reduced [45].

Behavioral interventions are also important. When AI-related anxiety leads to avoidance, patients may refrain from using AI tools or excessively avoid related information, further diminishing their long-term self-efficacy [46]. Therefore, an exposure-based approach involving the gradual use of AI tools may be helpful [47]. For example, by starting with relatively low-stress tasks, such as simple information searches, document summarization, or schedule organization, patients can experience a discrepancy between actual and anticipated risks while using AI. This process allows patients to confirm that their fear of AI is not an absolute fact but can be amplified by cognitive interpretation, thereby helping reduce avoidance and restore self-efficacy [43].

#### 3) Acceptance and commitment therapy

Acceptance and commitment therapy (ACT) focuses on addressing uncertainties and anxieties regarding the future, aspects that are within one’s control. ACT helps individuals move beyond the need to eliminate anxiety, enabling them to truly engage with their experiences and habitual thoughts while guiding them to act in alignment with their deeply held values [48].

ACT’s cognitive defusion helps individuals with AIA view thoughts, such as the belief that they will fail or become worthless, not as reality itself but as a single thought event arising in their minds [49]. Reframing the thought “I will fail because of AI” as “I am currently having the thought ‘I will fail because of AI’” creates psychological distance between the thought and the self. This distance minimizes anxious thoughts that dominate emotions and behaviors [50].

Mindfulness is another key technique used in ACT. This approach helps to reduce excessive cognitive and emotional reactions rather than prompting an immediate response to stimuli [51]. To achieve this, patients are trained to observe their physical sensations, emotions, and thoughts exactly as they are in situations that trigger AIA [52]. Accordingly, patients can alleviate anticipatory anxiety and maintain psychological flexibility even in uncertain situations.

Value-based behavior is particularly important in ACT because the more severe the AIA, the more patients may become preoccupied with reducing anxiety and avoiding important activities in their lives. The goal of therapy is not to create a safe state free from AIA but to help patients find a way of living that suits them, even in the presence of AIA [52,53]. For example, patients can explore questions such as, “What relationships and roles do I want to sustain even as AI advances?” and “What meaningful actions can I take today, even if I feel anxious?” This approach helps to reframe AIA from a struggle against an uncontrollable future into action aimed at living a meaningful life [51,54].

#### 4) Meaning-centered psychotherapy

Meaning-centered psychotherapy (MCP) addresses the loss of meaning in life, threats to professional identity, and existential instability, all of which are commonly observed in patients with AIA. MCP does not focus on eliminating these symptoms themselves but aims to alleviate emotional distress by helping individuals reconstruct their perceptions of life’s meaning and value [55].

The core of MCP lies in helping individuals move beyond a self-definition centered on their occupation or productivity to reconstructing values, relationships, attitudes, responsibilities, and unique life stories [56]. The belief that “if my job disappears, I become worthless,” commonly found among individuals experiencing AIA, is further reinforced when meaning is overly tied to a specific role or achievement. By deconstructing the equation “job equals existence” and guiding individuals to perceive their values and meaning in multiple dimensions, this approach helps ensure that AI-related role changes are not interpreted as a loss of identity [57]. For example, questions such as, “What is my role that AI cannot replace?” and “What attitude can I adopt even when facing an uncertain future?” can help reconstruct meaning. Such exploration ensures that AI-related role changes are not interpreted as a loss of existence and enables patients to maintain their self-determination and sense of life’s meaning, even in a changing environment.

#### 5) Digital well-being and lifestyle modification

Adjusting lifestyle habits and promoting digital well-being are crucial for AIA management [58]. Given that the duration of an individual’s exposure to information and technological stimuli can exacerbate AIA, psychiatric interventions should incorporate approaches that help patients modify their daily routines and relationships with technology.

First, it is necessary to set digital boundaries. These fundamental behavioral guidelines can be recommended for patients with AIA. Remaining constantly connected to chatbots, emails, AI-related news, and technology trends, even outside work hours, can lead to chronic anxiety and deprive patients of recovery time [59]. Therefore, patients can be guided to actively exercise their right to disconnect by intentionally blocking access to work-related messages or AI-related information after a certain time. By directly intervening in the core mechanism of AIA (i.e., the loss of a sense of control), such boundary setting helps individuals regain a sense of agency over their technological environment.

Second, the quality and quantity of AIA-related information must be regulated. When experiencing AIA, individuals may repeatedly search for AI-related information to reduce their anxiety; however, this can intensify their anxiety as they are exposed to catastrophic news or sensational content. Therefore, patients can be advised to select a few reliable sources of information, limit the time spent checking for updates, and avoid repetitive searches before bedtime or when anxiety is high. This helps to reduce compulsive checking behaviors and rumination.

Third, digital detox can reduce emotional hyperarousal by temporarily distancing oneself from technological stimuli. Patients are encouraged to allocate specific times to remain completely disconnected from smart devices and fill this time with offline activities, such as exercise, reading, outdoor activities, and face-to-face interactions [60]. Such experiences provide patients with physical and emotional experiences, emotional stability, and satisfaction, even without technology, and help alleviate the perception that AI dominates all areas of life.

Fourth, enhancing digital literacy is crucial for the prevention and treatment of AIA. AIA can stem from, or be exacerbated by, a lack of understanding of the technology’s actual limitations and operating principles. This is clearly demonstrated by research findings showing that higher AI literacy correlates with lower anxiety regarding AI, and that AIA mediates the relationship between literacy and attitudes [35]. Therefore, clinicians should guide individuals in learning, through trustworthy sources, about AI’s functional limitations, potential for errors, areas requiring human intervention, privacy protection methods, and ways to critically evaluate AI-generated results [61]. Such an approach helps individuals establish a more balanced relationship with AI technology.

#### 6) Strengthening human connection and care engagement

Maintaining interpersonal and actual caregiving relationships should not be overlooked in AIA management [62]. Social isolation and loneliness are significant mental health risk factors associated with depression, social anxiety, and suicidality [63], while social support can act as a protective factor that buffers stress responses [63]. Psychiatrists can help patients reestablish safe and trusting interpersonal relationships and, when necessary, utilize family counseling, group therapy, workplace support systems, and peer support programs.

When AIA is linked to occupational threats, individual therapy alone may be insufficient. Because job changes and employment insecurity resulting from AI implementation can affect an individual’s psychological safety and mental health, environmental interventions at the organizational level, such as job retraining, peer-based learning, organizational support, communication with managers, and job adjustments, should also be considered [64]. This is important for reframing AIA not merely as an individual vulnerability but also as an issue of organizational adaptation to sociotechnical changes and the protection of mental health.

### 7. Limitations and future directions

Although interest in AIA has recently increased, several significant limitations hinder its interpretation from a psychiatric perspective. First, its conceptual boundaries remain unclear because AIA overlaps with technostress, job insecurity, digital anxiety, and concerns regarding human–machine relationships [10,65,66]. Further research is needed to define AIA as a distinct or transdiagnostic stress-related construct and to clarify its role in psychiatric assessment and formulation.

Second, existing studies focused on students, workers, and technology users, thereby limiting their generalizability. Population-based research should examine prevalence, vulnerable groups, and protective factors across demographic, socioeconomic, occupational, and clinical characteristics.

Third, most studies used cross-sectional, self-report designs, which hinder the establishment of causal relationships between AIA and psychiatric symptoms. Longitudinal, repeated-measures, ecological momentary assessment, and clinical interview studies are required.

Fourth, the research on AIA in patients with psychiatric disorders remains limited. Specifically, further studies are warranted to examine the relationship between AIA and GenAI use, and the pursuit of reassurance, social isolation, delusion-like experiences, disconnection from others, and suicidal thoughts.

Finally, interventional studies on AIA are limited. Thus, intervention methods must be developed and validated to determine the effectiveness of psychoeducation, cognitive and behavioral approaches, value- and meaning-based interventions, and digital boundary settings across various clinical populations.

## Conclusion

Throughout human history, technological changes have consistently affected mental health. However, AI uniquely mimics the human mind, resulting in exceptionally far-reaching consequences. Psychiatry is well-suited to address this issue, as the field possesses the expertise to understand AI within the context of rapid sociotechnological change and to distinguish between normal stress responses and conditions requiring clinical intervention.

This review conceptualizes AIA as a multidimensional psychological response arising from the interaction of complex factors. From this perspective, AIA should be viewed as a sociopsychiatric phenomenon shaped by technological shifts, labor market dynamics, social trust, digital culture, and changing human relationships.

Although current research suggests a link between AIA and anxiety and depressive symptoms, studies regarding causality or long-term clinical outcomes remain limited. Therefore, rather than prematurely classifying AIA as an independent mental disorder, viewing it as an AI-related stress response that can trigger or exacerbate existing mental health conditions is more appropriate. From a clinical perspective, it is essential to carefully evaluate whether AI-related anxiety represents a transient concern, an adjustment issue accompanied by functional impairment, an exacerbation of pre-existing anxiety or depression, or a condition linked to factors such as obsessive rumination, delusion-like experiences, or avoidance of human caregiving relationships.

Psychiatric interventions must go beyond merely alleviating anxiety to help patients regain a sense of agency, self-efficacy, meaning in life, and human connections amid rapid technological changes. This requires an integrated approach encompassing psychoeducation, cognitive restructuring, acceptance and values-based behavior, meaning-centered approaches, digital well-being, and strengthening human care relationships. This literature review may serve as a starting point for the psychiatric discourse aimed at understanding and protecting the mental health of the public and patients during the era of AI.

## Figures and Tables

**Fig. 1.**
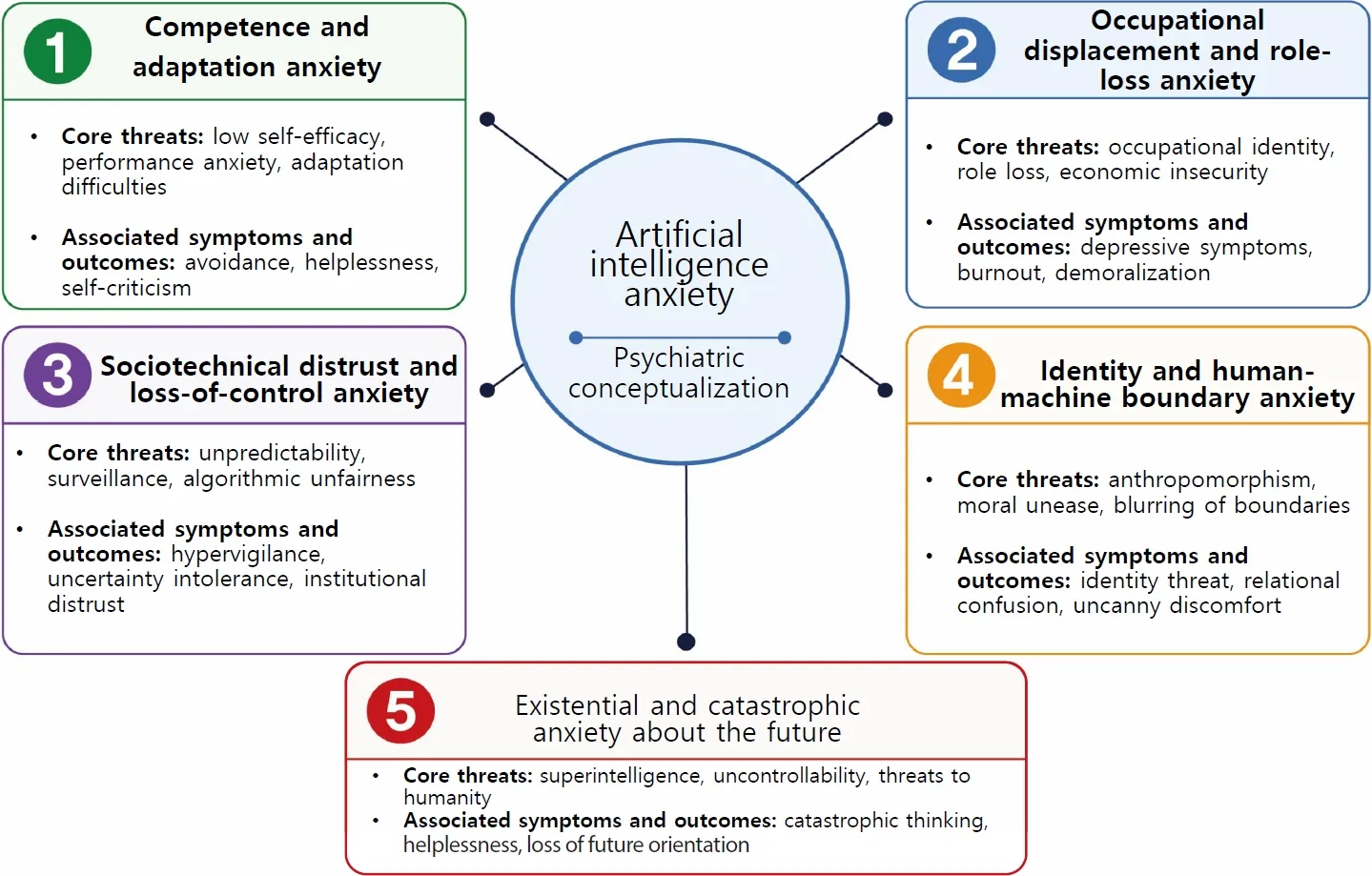
Psychiatric conceptualization of artificial intelligence anxiety. These five domains are conceptually distinct, but they may also interact with and reinforce one another.

**Table 1.** Clinical interventions for AIA and their key therapeutic principles

Intervention	Key therapeutic concept
Psychoeducation	It explains AIA as a stress response resulting from rapid sociotechnical changes. While normalizing the patient’s distress, patients are educated on how excessive anxiety can lead to avoidance, repetitive checking behaviors, sleep disturbances, feelings of depression, and social isolation
Cognitive behavioral therapy	It addresses catastrophic thinking, overestimation of probability, dichotomous thinking, selective attention, and confirmation bias that characterize AIA. It identifies automatic thoughts that interpret AI as an all-encompassing threat and helps patients reframe them into more balanced thinking. If necessary, an exposure-based approach can be used, in which patients gradually try using AI tools in a controlled environment
Acceptance and commitment therapy	Rather than seeking to completely eliminate an uncontrollable future and uncertainty, this approach helps individuals recognize and accept uncomfortable thoughts and emotions related to AI while enabling them to act in accordance with the values they hold dear. Through cognitive defusion, mindfulness, acceptance, and value-based action, it reduces rumination and avoidance and increases psychological flexibility
Meaning-centered psychotherapy	It helps individuals move beyond self-definitions centered on careers or productivity to the reconstruction of values, relationships, responsibilities, and unique life stories. It mitigates the perception that one’s career defines one’s very existence and ensures that AI-related role changes are not interpreted as a loss of identity
Digital boundary setting	It reduces the amount of time spent constantly engaging with chatbots, emails, AI-related news, and technology trends outside of work hours. Limiting access to AI-related information after a certain time and reducing digital stimulation before bed help restore a sense of control and allows for recovery time
Digital detox	It helps people spend a certain period each day or part of the weekend disconnected from their smart devices. Offline activities such as exercise, reading, spending time in nature, and face-to-face interactions enable people to experience emotional stability and satisfaction without relying on technology
Digital literacy enhancement	It helps individuals learn about the functional limitations of AI, potential for errors, areas requiring human intervention, methods for protecting personal information, and critical evaluation of AI results. This helps mitigate the tendency to perceive AI as either all-powerful or uncontrollable and enables realistic risk assessments
Strengthening human connection and engagement	It assesses whether AI use complements or replaces human relationships. It views avoidance of real-life interpersonal or therapeutic relationships as a warning sign of human care disengagement and helps individuals restore genuine caring relationships with family, friends, colleagues, and therapists

AIA, artificial intelligence anxiety; AI, artificial intelligence.
